# Potentially harmful excipients in drugs: an analysis of labeling practices and adverse drug reactions

**DOI:** 10.3389/fphar.2026.1949057

**Published:** 2026-10-13

**Authors:** Su-ping Li, Xia-yang Wu, Jun-kai Ning, Xiao-qing Huang, Yi-juan Shen, Yu-ying Chen, Kai-jun Liao, Feng Weng, You-hong Wang

**Affiliations:** 1 Department of Pharmacy, Children’s Hospital of Fudan University (Xiamen Branch), Xiamen Children’s Hospital, Xiamen, China; 2 Department of Pharmacy, Quanzhou Women’s and Children’s Hospital, Quanzhou, China; 3 Department of Pharmacy, Women and Children’s Hospital Affiliated to Xiamen University, Xiamen, China; 4 Department of Pharmacy, Zhongshan Hospital Affiliated to Xiamen University, Xiamen, China; 5 Department of Pharmacy, Longyan First Affiliated Hospital of Fujian Medical University, Longyan, China; 6 Department of Pharmacy, Nanping First Hospital Affiliated to Fujian Medical University, Nanping, China; 7 Department of Pharmacy, Fuzhou Children’s Hospital of Fujian Province, Fuzhou, China; 8 Fujian Key Laboratory of Neonatal Diseases, Xiamen Key Laboratory of Neonatal Diseases, Xiamen Children’s Hospital (Children’s Hospital of Fudan University at Xiamen), Xiamen, China

**Keywords:** adverse drug reactions, drug labeling, excipients, pediatric, reporting odds ratio

## Abstract

**Background:**

Excipients play a crucial role in pharmaceutical formulations, however, their safety profile in pediatric populations remains inadequately characterized.

**Methods:**

This retrospective multicenter study was conducted in two phases. Phase one reviewed package inserts of pediatric medications used at seven Fujian hospitals (July 2019–June 2024) to assess excipient labeling. Phase two analyzed associations between potentially harmful excipients (PHEs) exposure and adverse drug reactions using the reporting odds ratio (ROR) method at a single children's hospital.

**Results:**

The rates of excipient labeling exhibited significant variation based on hospital type, drug source, and drug category. In total, 41.3% of pediatric cases and 43.2% of prescriptions included at least one PHE. Benzyl alcohol was associated with application site pain (ROR = 393.20, *P* < 0.05) and erythema (ROR = 85.84, *P* < 0.05); thimerosal was linked to application site irritation (ROR = 278.35, *P* < 0.05) and pruritus (ROR = 115.20, *P* < 0.05); and sodium citrate was associated with bradycardia (ROR = 157.59, *P* < 0.05).

**Conclusion:**

The labeling of excipients in pediatric pharmaceutical products is insufficient in the institutions examined, potentially hindering the identification of excipient exposure in clinical practice. The safety signals identified in this study necessitate further systematic investigation and careful consideration within both regulatory and clinical contexts.

## 1 Introduction

Pediatric medication safety is a long-standing core issue in the field of pediatric pharmacotherapy. Compared with adults, children, especially neonates and infants, exhibit significant differences in pharmacokinetics, organ maturation, and physiological compensatory capacity, making them more susceptible to adverse drug reactions (including those from active pharmaceutical ingredients and excipients) (Kozer et al., 2006; Ghaleb et al., 2006; Hughes and Edgerton, 2005). Excipients play a crucial role in medicinal products by enhancing solubility, stability, bioavailability, and palatability of dosage forms (Salunke et al., 2017). Despite their widespread use and general perception of safety, the safety profile of excipients in vulnerable populations, particularly children, remains inadequately characterized (Allegaert and van den Anker, 2015). In contrast to active pharmaceutical ingredients, excipients are frequently regarded as “inert”; however, emerging evidence suggests that certain excipients may pose risks to neonates and young children due to factors such as immature metabolic pathways, prolonged exposure, and cumulative dosing (Salunke et al., 2017; Turner et al., 2014; Whittaker et al., 2009). Several excipients have been associated with adverse events in pediatric populations (Hall et al., 2004; Pesce and McKean, 1989; Collison et al., 2012). A recent systematic review identified 54 excipients with potential toxicity in children and classified them into four functional categories, emphasizing that their adverse effects include hepatotoxicity, nephrotoxicity, neurotoxicity, and hypersensitivity reactions, with higher risk in younger age groups (Silva-Chocoteco et al., 2026).

The susceptibility of children to excipient-related toxicity is heightened due to the dependence on excipient labeling and safety data predominantly derived from adult populations (Bobillot et al., 2024; American Academy of Pediatrics Committee on Drugs, 1984; Comoglu and Ozyilmaz, 2025). Pediatric patients exhibit notable differences in drug metabolism, organ maturation, and body composition, which can significantly affect excipient pharmacokinetics and toxicity thresholds (Kearns et al., 2003). Currently, international regulatory bodies such as the U.S. Food and Drug Administration (FDA), the European Medicines Agency (EMA), and the International Council for Harmonisation of Technical Requirements for Pharmaceuticals for Human Use (ICH) have issued lists or guidelines identifying several excipients requiring special attention and have established daily acceptable intake levels or safety thresholds for them (Silva-Chocoteco et al., 2026). However, the applicability of these standards varies across regions and dosage forms, and systematic comprehensive assessments specifically for the pediatric population are lacking (Silva-Chocoteco et al., 2026). In clinical practice, although studies such as the SEEN project have explicitly planned to quantify cumulative exposure to nine potentially harmful excipients (including ethanol, propylene glycol, and benzyl alcohol) in Danish neonates and children under 5 years of age (Valeur et al., 2017), large-scale, multicenter exposure data are still insufficient to systematically reveal the prevalence and dose characteristics of excipient exposure in children, especially those receiving multiple medications.

International research indicates that exposure to harmful excipients is prevalent among neonatal and pediatric populations. A pan-European study encompassing 89 neonatal units found that 43% of neonates received at least one potentially harmful excipient (Nellis et al., 2015). Similarly, research conducted in Estonia and Brazil showed that nearly all hospitalized neonates were exposed to excipients with established toxicity profiles (Lass et al., 2012; Souza et al., 2014). Valeur et al. reported that the daily exposure of neonates to ethanol and propylene glycol frequently exceeds the EMA’s tolerance limits by factors of 261 and 19, respectively (Valeur et al., 2018). Conversely, the annotation of excipient ingredients, quantities, and warning information in drug instructions and labels is inconsistent and inadequate on a global scale (Silva-Chocoteco et al., 2026). This lack of information hinders clinicians’ ability to accurately identify the types and doses of excipients to which patients are exposed during prescribing, dispensing, and administration, thereby impeding the effective assessment and mitigation of potential risks. When exposure data cannot be matched with available labeling information, the risk identification chain is broken—the risks revealed by exposure evidence cannot be translated into specific actions in clinical decision-making, while incomplete labeling information further obscures risks, thereby creating potential points of vulnerability in pediatric medication safety.

In this context, our study integrates multicenter data on excipient labeling with real-world analyses of adverse events to: (1) systematically characterize excipient labeling and usage patterns in pediatric medications across Fujian Province; (2) identify commonly used excipients that may pose potential harm to pediatric populations; and (3) detect safety signals for specific excipients and adverse events using the ROR method. Our study employs a “labeling–exposure–risk” analytical framework with the objective of summarizing the exposure patterns of potentially harmful excipients in pediatric medications, examining the evidence connecting these excipients to the risk of adverse events, and advocating for safer medication practices in pediatric populations.

## 2 Materials and methods

### 2.1 Study design and data collection

A retrospective observational design was employed to assess pediatric excipient exposure in Fujian Province. The research process comprised two distinct stages. In the first stage, excipient data were collected from pediatric medications used in seven hospitals within Fujian Province. Drug formularies from July 2019 to June 2024 were reviewed to obtain package inserts for pediatric drugs prescribed in both inpatient and outpatient settings. For each pharmaceutical agent, data were systematically collected concerning its origin (imported or domestic), classification (Chinese patent medicine or Western medicine), dosage form, disclosure status of excipients, types of excipients, identification of harmful or potentially harmful excipients, excipient content when available, and any warnings or precautions specific to pediatric use. In the subsequent phase, a retrospective chart review was conducted at pediatric hospitals to investigate the association between exposure to PHE and ADRs. Data on ADRs were sourced from the Adverse Drug Reaction Monitoring System from July 2019 to June 2024. The inclusion criteria were as follows: (1) patients aged 0 to 18 years; (2) documented exposure to the target excipient in medical or prescription records, with exposure to at least one formulation containing the excipient during hospitalization. The exclusion criteria were: (1) use of the excipient as a therapeutic agent; (2) incomplete or missing records regarding excipient exposure; (3) administration limited to electrolytes or dissolving solutions; (4) severe hepatic or renal impairment that could influence excipient metabolism or elimination.

### 2.2 Ethics approval and consent statement

The study protocol was approved by the Ethics Committee of Xiamen Children’s Hospital (Approval No. 2024124).

### 2.3 Medication exposure and ADR records

Each patient was identified by a unique hospital identifier, allowing for the linkage of medication exposure records to ADR reports via patient ID. An event was classified as exposure-associated only if the medication administration date preceded the ADR report date. Suspected drugs were initially identified by the attending physician or clinical pharmacist in the ADR report and subsequently verified by a second pharmacist. The analytical unit for the disproportionality analysis was the patient–ADR observation recorded in the hospital’s Adverse Drug Reaction Monitoring System. Each patient was counted no more than once within any specific excipient–ADR pair. However, patients with multiple adverse events or those receiving multiple medications containing the same excipient could contribute to more than one pair. Consequently, the total number of observations used to construct the 2 × 2 contingency tables (n = 4,864,817) exceeds the number of unique pediatric patients (n = 3,268,706).

### 2.4 Identification of potentially harmful excipients and data collection

We conducted a systematic literature review to identify excipients reported as potentially harmful to pediatric populations. PHEs are defined as those excipients that have been identified and marked in the scientific literature as posing potential safety concerns or adverse effects following exposure. Research findings were searched from PubMed. The PubMed search strategy used the following terms: ((“Child”[Mesh] OR “Child, Preschool”[Mesh] OR “Infant”[Mesh] OR “Adolescent”[Mesh]) OR (child*[tiab] OR pediatric*[tiab] OR paediatric*[tiab] OR infant*[tiab] OR neonat*[tiab] OR adolescent*[tiab])) AND ((“Excipients”[Mesh] OR “Pharmaceutic Aids”[Mesh]) OR (excipient*[tiab] OR “pharmaceutic aid*”[tiab] OR “inactive ingredient*”[tiab])). Initially, we conducted a screening of titles and abstracts to assess their potential relevance. This process involved an independent review of titles by two authors, with any disagreements being resolved through consultation with a third author. Following this, we retrieved full-text articles that were considered relevant. Our search imposed no restrictions on publication dates, methodologies, or study types, but was confined to articles published in English. Studies were excluded if full-text articles were not accessible. Supplementary Table S1 provides a summary of adverse events and corresponding references for these PHEs.

### 2.5 Statistical analysis

All statistical analyses were executed using SPSS version 27 (IBM Corp.). Descriptive statistics were employed to summarize the excipient labeling status across seven medical institutions. Continuous variables are presented as either mean ± standard deviation or median with interquartile range, as appropriate. Categorical variables are expressed as frequencies and percentages. The chi-square test was used to compare the PHE exposure frequency between patients with and without adverse events, as well as to compare excipient labeling rates and usage frequencies across groups; Fisher’s exact test was applied when the expected count in any cell was less than 5. All statistical tests were two-sided, with *P* values less than 0.05 considered statistically significant.

In pharmacovigilance research, disproportionality analysis is employed to evaluate the relative frequency of specific ADRs associated with one or more drugs, in comparison to the frequency of ADRs for the same drug reported across the entire database. Signal detection is predicated on a 2 × 2 contingency table utilized for disproportionality analysis: ‘a’ denotes the number of reports containing both the target drug and the target adverse reactions; ‘b’ represents the number of reports containing the target drug with other ADRs; ‘c’ indicates the number of reports containing other drugs with the target ADRs; and ‘d’ signifies the number of reports containing both other drugs and other ADRs. The ROR serves as a statistical measure in pharmacovigilance to evaluate the association between a specific ADRs and a specific drug, and to contrast it with all other drugs in the database. The formula for calculating the ROR is as follows:

$$\text{ROR}=\frac{\text{ad}}{\text{bc}}$$

$$95\%\text{CI}=e^{\ln\left(\text{ROR}\right)\pm 1.96\sqrt{\left(\frac{1}{a}+\frac{1}{b}+\frac{1}{c}+\frac{1}{d}\right)}}$$

$$x^{2}=\left[{\left(ad-bc\right)}^{2}\right]*\left(a+b+c+d\right)/\left[\left(a+b\right)\left(c+d\right)\left(a+c\right)\left(b+d\right)\right]$$

A positive ROR signal was defined *a priori* as at least three reports containing both the target excipient and the target adverse event (a ≥ 3) and a lower bound of the 95% CI of the ROR greater than 1. Associations with ROR ≥2 were considered robust signals, whereas associations with 1 < ROR <2 were considered weak. Anchor the twofold threshold in the established signal-screening criterion for spontaneous-report disproportionality analyses (PRR ≥2 with χ^2^ ≥ 4 and a minimum of three cases, as per Evans et al., 2001) (Evans et al., 2001), a criterion that has been extensively adapted for ROR-based analyses of large pharmacovigilance databases. RORs and their 95% CIs were estimated using Woolf’s (logit) method. To address instances where some cell values equaled zero in the 2 × 2 contingency table, the Haldane-Anscombe correction (addition of 0.5 to each cell) was applied prior to calculation. The corrected formula for the ROR is as follows:

$$\text{ROR}=\frac{\left(a+0.5\right)\times \left(d+0.5\right)}{\left(b+0.5\right)\times \left(c+0.5\right)}$$

Because a large number of excipient–ADR associations were evaluated, a Benjamini–Hochberg false discovery rate (FDR) adjustment was performed as a sensitivity analysis, using the exact *P* values of the chi-square tests (df = 1) for the 99 associations tested (i.e., all excipient–ADR pairs with at least three reports, a ≥3). The 99 *P* values were ranked in ascending order (p(1) ≤ p(2) ≤ … ≤ p(99)); the *i*th smallest P value was considered significant at FDR q < 0.05 if p(i) ≤ 0.05·i/99, and FDR-adjusted P values (q values) were computed as q(i) = min_{j ≥ i} {p(j)·99/j}. For associations with zero cells in the 2 × 2 table, 0.5 was added to each cell (Haldane–Anscombe correction) before calculation. FDR-adjusted q values for all associations are provided in Supplementary Table S2.

## 3 Results

### 3.1 General information on excipient labeling

Excipient ingredient labeling rates in seven Fujian Province medical institutions are consistently high, between 47.4% and 54.3%, with imported drugs leading, followed by domestic and Western medicines. Chinese patent medicines show the most variability and lower rates. Excipient content labeling rates are low, ranging from 3.36% to 4.64%, with specialized hospitals generally performing better than general hospitals (Figure 1).

**FIGURE 1:**
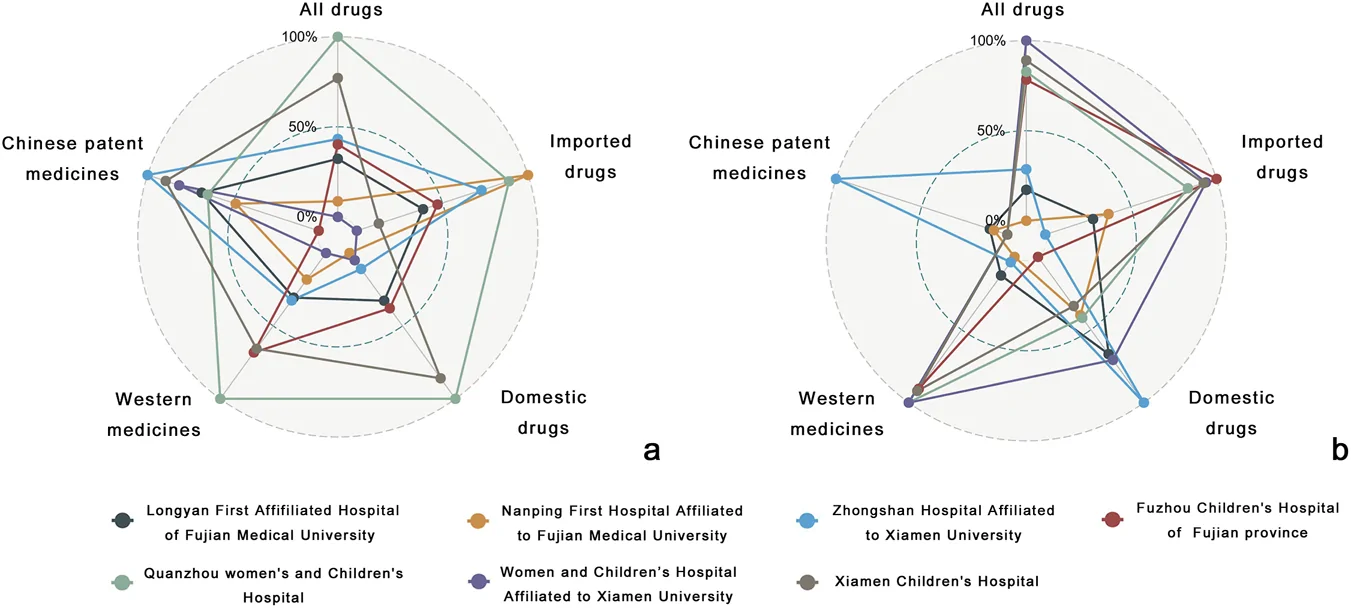
Radar charts illustrating pharmaceutical excipient labeling rates across seven medical institutions in Fujian Province. **(a)** Excipient ingredient labeling rates. **(b)** Excipient content labeling rates.

The rates of excipient labeling exhibited significant variation based on hospital type, drug source, and drug category (Table 1). Specialized hospitals shown a labeling rate of 51.8%, which was notably higher than the 49.3% observed in general hospitals (*P* < 0.05). Additionally, imported products exhibited a 1.25-fold higher labeling rate than domestic products (*P* < 0.001). Western medicines were labeled at significantly higher rates than Chinese patent medicines (51.3% vs. 39.3%, *P* < 0.001). However, for excipient content labeling, the overall rates remained below 5% across both hospital types. The labeling rate for imported products stood at 11.1%, which was 5.8 times higher than that for domestic products (χ^2^ = 248.361, *P* < 0.001). Western medicines had a labeling rate of 4.2%, significantly exceeding the 0.6% rate for Chinese patent medicines, representing an approximately 7.0-fold difference (χ^2^ = 16.909, *P* < 0.001), as shown in Table 2.

**TABLE 1:** Excipient labeling rates stratified by hospital type, drug source, and drug classification.

Comparison	Labeling rate (%)	χ^2^	*P*-value
Hospital type			
General hospitals	49.3	3.950	<0.05
Specialized hospitals	51.8	​	​
Drug source			
Imported drugs	59.3	61.845	<0.001
Domestic drugs	47.6	​	​
Drug type			
Western medicines	51.3	27.649	<0.001
Chinese patent medicine	39.3		

**TABLE 2:** Excipient content labeling rates stratified by hospital type, drug source, and drug classification.

Comparison	Labeling rate (%)	χ^2^	*P*-value
Hospital type			
General hospitals	3.6	3.557	>0.05
Specialized hospitals	4.5	​	​
Drug source			
Imported drugs	11.1	248.361	<0.001
Domestic drugs	1.9	​	​
Drug type			
Western medicines	4.2	16.909	<0.001
Chinese patent medicine	0.6		

### 3.2 Excipient labeling rates stratified by drug formulations

The rates of excipient labeling demonstrated significant variability across different pharmaceutical dosage forms, with a range from 11.8% to 83.3% (Figure 2). The highest labeling rates were observed in implants (83.3%), injections (80.3%), and eye drops (67.7%), while ear drops exhibited the lowest rate (11.8%). Within oral solid dosage forms, powders and oral powders had relatively high labeling rates, whereas tablets showed the lowest rate. A stratified analysis by hospital type revealed that for most dosage forms, excipient labeling rates were broadly similar between general and specialized hospitals, with notable exceptions: granules had a higher labeling rate in specialized hospitals (47.0%) compared to general hospitals (32.35%), whereas topical solutions were more frequently labeled in general hospitals (32.61%) than in specialized hospitals (20.75%) (Figure 3).

**FIGURE 2:**
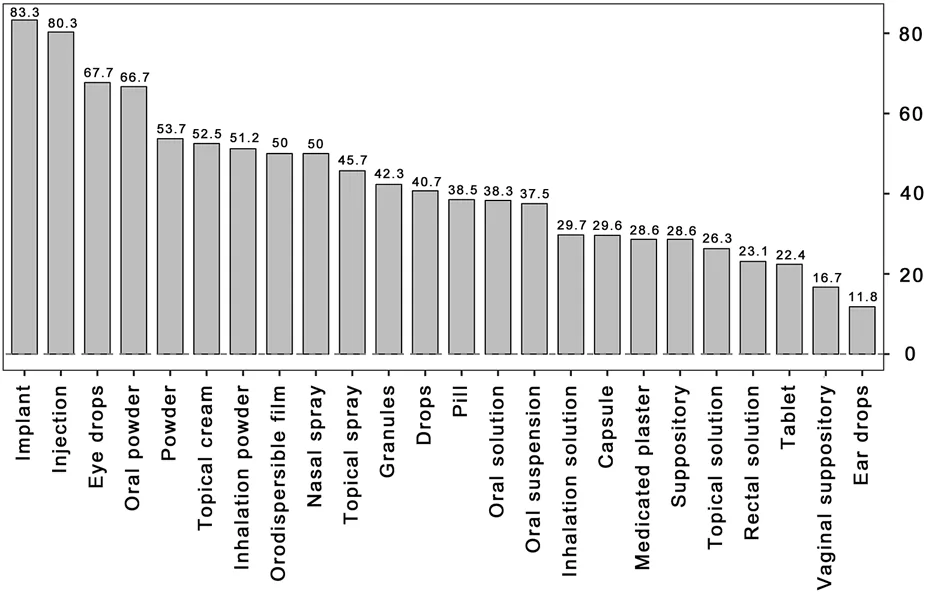
Ranking of 24 pharmaceutical dosage forms by excipient ingredient labeling rate (%). The labeling rate indicates the percentage of products within each dosage form that disclose excipient ingredients in their product specifications.

**FIGURE 3:**
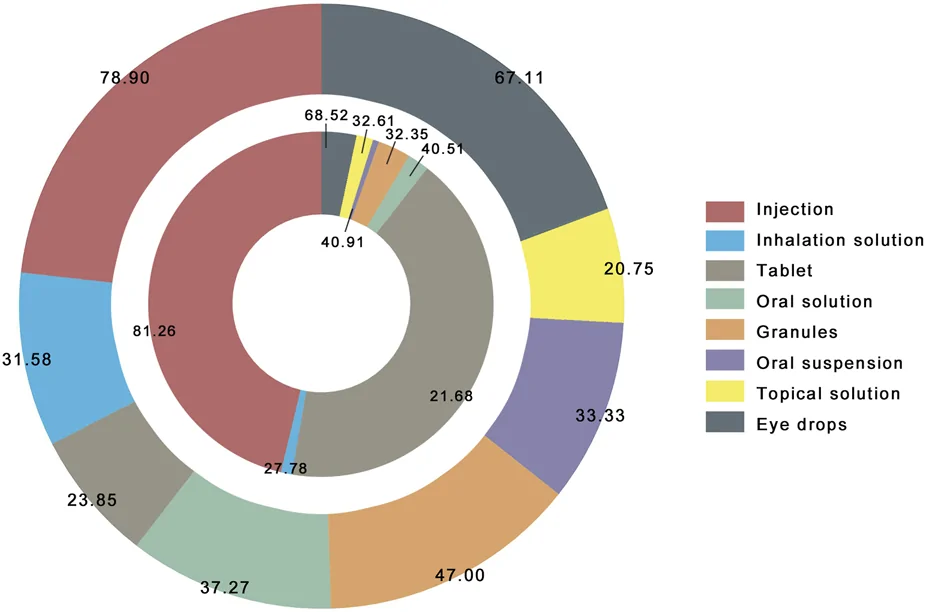
Double-layer donut chart comparing excipient ingredient labeling rates for commonly used pediatric dosage forms between general hospitals and specialized hospitals. The inner ring color shows the labeling rate (%) for each dosage form in general hospitals, and the arc width represents its proportion among commonly used formulations in these hospitals. The outer ring color shows the labeling rate (%) for each dosage form in specialized hospitals, and the arc width represents its proportion among commonly used formulations in these hospitals. Data are based on product specification analysis of pediatric medications.

### 3.3 Comparison of excipient usage frequency between hospital types

The study conducted an analysis of the usage frequency of 34 excipients across 11 functional categories in pediatric formulations, comparing their application in general hospitals versus specialized hospitals. Among these excipients, 10 (29.4%) exhibited statistically significant differences in usage (*P* < 0.05) (Table 3). Notably, lactose showed the most significant disparity, with a substantially higher usage rate in general hospitals compared to specialized hospitals (7.1% vs. 3.6%, χ^2^ = 35.192, *P* < 0.001). In contrast, specialized hospitals demonstrated higher usage rates of several antimicrobial preservatives, including benzyl alcohol, sodium benzoate, ethylparaben, metacresol, and sulfites (*P* < 0.05). Additionally, the sweetener sucrose was utilized at a significantly higher rate in specialized hospitals (5.9% vs. 3.3%, χ^2^ = 27.363, *P* < 0.001).

**TABLE 3:** Frequency comparison of excipient usage in general hospitals and specialized hospitals.

Excipient category	Specific function	Excipient	Frequency of excipient use (%)		χ^2^	*P*-value
			General hospitals	Specialized hospitals		
Antimicrobial preservatives	Antimicrobial, solvent	Benzyl alcohol	0.7	1.3	6.622	***P* < 0.05**
	Antimicrobial, antiseptic, solubilising, wetting	Benzalkonium chloride	1.5	2	2.626	*P* > 0.05
	Preservative, antimicrobial, tablet/capsule lubricant	Sodium benzoate	0.9	1.6	7.472	***P* < 0.05**
	Antimicrobial preservatives	Benzoic acid	0.3	0.5	1.261	*P* > 0.05
	Antimicrobial preservative and disinfectant	Phenol	2.3	1.9	1.064	*P* > 0.05
	Antimicrobial preservative; disinfectant	Metacresol	0	0.2	7.131	***P *< 0.05**
	Preservative	Thimerosal	0.2	0.2	0.001	*P* > 0.05
	Preservative	Ethylparaben	0.8	1.4	5.511	***P* < 0.05**
	Preservative	Methylparaben	0.2	0.3	0.239	*P* > 0.05
	Preservative	Propylparaben	0.2	0.2	0.381	*P* > 0.05
	Solvent/preservative	Ethanol/ethyl alcohol	2.7	2.9	0.105	*P* > 0.05
	Antimicrobial (antimicrobial preservative)	Sodium metabisulfite	1.4	1.5	0.224	*P* > 0.05
	Antioxidant, antimicrobial preservative	Sulfites	2.5	3.6	7.107	***P* < 0.05**
Solvents/Cosolvents	Antimicrobial, humectant, plasticizer, solvent, stabilizing, water-miscible cosolvent, humectant	Propylene glycol	1.7	2.3	2.470	*P* > 0.05
	Solvent/humectant	Glycerol	4.6	5.7	3.637	*P* > 0.05
	Ointment base; plasticizer; solvent; suppository base	Polyethylene glycol	1.6	1.8	0.395	*P* > 0.05
	Emollient; oleaginous vehicle; solvent, emulsifying agent; solubilising agent and wetting agent	Polyoxyethylene castor oil	0.8	0.5	2.229	*P* > 0.05
Surfactants/Emulsifiers/Solubilizers	Dispersing, emulsifying, non-ionic surfactant, solubilising, suspending, wetting	80 polysorbate	4.6	3.9	1.616	*P* > 0.05
Fillers/Diluents/Direct compression excipients	Bulking agen, directly compressible tablet excipient; tablet and capsule diluent; tablet and capsule filler	Lactose	7.1	3.6	35.192	***P *< 0.001**
	Diluent; plasticizer; sweetening agent; tablet and capsule diluent; therapeutic agent; and tonicity agent	Mannitol	6.2	4.8	5.695	***P* < 0.05**
Sweetening agents	Sweetening agent	Saccharin	0.3	0.7	4.380	***P* < 0.05**
	Sweetening agent	Sodium saccharine	0.4	0.7	3.655	*P* > 0.05
	Sweetening agent	Sucrose	3.3	5.9	27.363	***P* < 0.001**
	Sweetening agent/viscosity agent	Sorbitol	1.6	1.3	0.561	*P* > 0.05
Coloring agents/Dyes	Coloring agents/dyes	Tartrazine	0.3	0.3	0	*P* > 0.05
	Coloring agents/dyes	Sunset yellow	0.1	0	0.077	*P* > 0.05
	Colourant	Indigo	0.1	0	1.404	*P* > 0.05
	Colourant	Carmine	0.2	0.4	3.780	*P* > 0.05
Viscosity increasing agents/Suspending agents/Stabilizers	Viscosity increasing agent/emulsifying/suspending	Xanthan gum	0.2	0.4	2.20	*P* > 0.05
	Viscosity increasing agent/emulsifying/suspending	Colloidal silicon dioxide	2.1	1.1	8.250	***P *< 0.05**
pH adjusters/Buffering agents	Acidifying agent	Acetic acid	0.8	1	0.952	*P* > 0.05
	Buffering agent/preservative	Citric acid	0.7	0.6	0.720	*P* > 0.05
Chelating agents	Chelating agent	Edetate disodium	3.9	4.7	2.372	*P* > 0.05
Coating agents/opacifiers	Coating as opacifier, pigment	Titanium dioxide	1.1	1	0.489	*P* > 0.05

Bold values indicate statistically significant differences (*P* < 0.05).

### 3.4 Regional variation in excipient labeling rates of imported pharmaceuticals by country of origin

Considerable geographic heterogeneity was observed in excipient labeling rates among imported pharmaceuticals by country of origin (Figure 4). In European countries, the highest rates of excipient labeling were recorded, notably in Norway (100%), Belgium (92.4%), Austria (87.5%), and Denmark (83.8%). Most Western European nations reported labeling rates exceeding 50%, whereas Eastern European countries demonstrated significantly lower rates. In North America, excipient labeling rates were generally moderate to high, with Canada (85.7%) and the United States (64.9%) ranking fourth and 12th, respectively. Conversely, Asian countries displayed relatively low excipient labeling rates overall, with seven countries, including South Korea, Thailand, Singapore, India, Mexico, Cyprus, and Poland, reporting no excipient labeling (0%).

**FIGURE 4:**
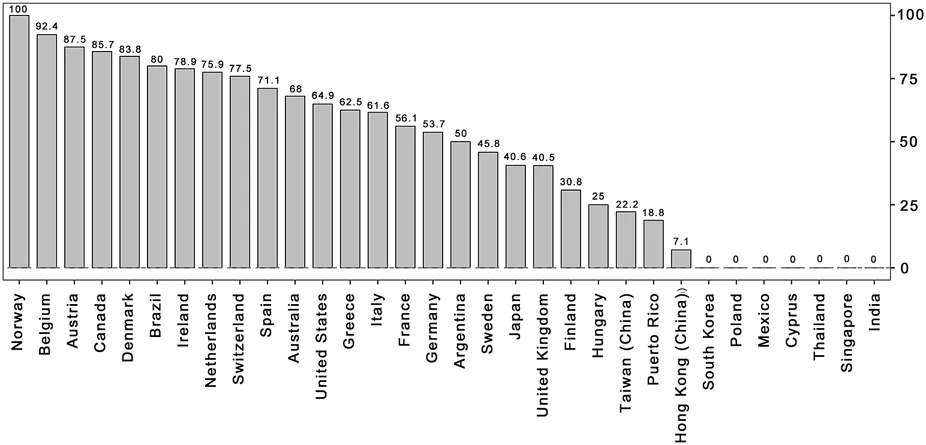
Excipient labeling rates of imported drugs in Fujian Province, ranked by source country.

### 3.5 Study population and overview of ADRs

Table 4 delineates the baseline characteristics and exposure to PHEs within the study cohort. The analysis encompassed 3,268,706 pediatric cases, comprising 58.8% males and 41.2% females, with a median age of 5.51 years. A total of 155 pharmaceuticals containing PHEs were identified, accounting for 24.4% of all medications. Overall, 41.3% of the cases were exposed to at least one PHE-containing pharmaceutical.

**TABLE 4:** Baseline characteristics of the study population and PHE exposure status.

Characteristics	No
Age (median, IQR)	5.51 (IQR: 2.88-8.66)
Gender	3268706
Male	1922484
Female	1346222
Prescriptions per case (median, IQR)	8.05 (IQR:5.5-12.5)
Number of drugs containing PHEs (%)	155 (24.4%)
Number of cases exposed to PHEs (%)	1349976 (41.3%)
Number of prescriptions containing PHEs (%)	11381655 (43.2%)

PHEs, potentially harmful excipients; IQR, interquartile range.

ADRs were classified into 14 System Organ Class (SOC) categories, totaling 51 documented ADRs (Table 5). Gastrointestinal disorders accounted for 30.42% of the cases, followed by skin and subcutaneous tissue disorders, and general disorders and administration site conditions. The predominant gastrointestinal events included diarrhea (2,372 cases), nausea (1,326 cases), vomiting (1,326 cases), and lactose intolerance (1,109 cases). Within the category of skin and subcutaneous tissue disorders, erythema, pruritus, and rash were noted, ranking second, fifth, and twelfth, respectively. General disorders and administration site conditions were the third most prevalent category, with pyrexia (2,044 cases) being the most common event.

**TABLE 5:** Distribution of adverse events by SOC.

System organ class	No (%)	Adverse drug reactions	No	Rank
Immune system disorders	2893 (10.07)	Allergic reaction	2893	1
Vascular disorders	121 (0.42)	Phlebitis	121	41
Skin and subcutaneous tissue disorders	5384 (18.75)	Erythema Pruritus Rash Flushing	2552 1389 812 631	2 5 12 16
Respiratory, thoracic and mediastinal disorders	684 (2.38)	Cough	684	14
Psychiatric disorders	1406 (4.9)	Dysphoria Stress Anxiety Sleep disorder	667 325 220 194	15 24 29 31
Nervous system disorders	2224 (7.74)	Dizziness Migraine Somnolence Taste disorder Vertigo	913 554 179 139 439	10 18 32 36 21
Metabolism and nutrition disorders	334 (1.16)	Decreased appetite Hypocalcaemia	247 87	27 48
Hepatobiliary disorders	163 (0.57)	Jaundice	163	33
General disorders and administration site conditions	3879 (13.51)	Fatigue Chills Pyrexia Application site pain Application site irritation Injection site swelling Thirst Oedema	401 123 2044 523 324 209 146 109	22 40 4 19 25 30 34 43
Gastrointestinal disorders	8736 (30.42)	Diarrhoea Nausea Vomiting Lactose intolerance Abdominal discomfort Malabsorption Constipation Dyspepsia	2372 1326 1326 1109 980 883 598 142	3 6 7 8 9 11 17 35
Eye disorders	1699 (5.92)	Eye irritation Ocular hyperaemia Eyelid oedema Conjunctivitis Eye pain Vision blurred Eye pruritus Eye infection	695 328 221 127 103 89 74 62	13 23 28 39 44 45 49 50
Cardiac disorders	636 (2.21)	Palpitations Bradycardia Tachycardia Arrhythmia	250 136 131 119	26 37 38 42
Blood and lymphatic system disorders	538 (1.87)	Neutropenia Pancytopenia	449 89	20 45
Injury, poisoning and procedural complications	25 (0.09)	Asphyxia	25	51

### 3.6 Association of specific excipients with ADRs

We analyzed the signals in Table 6 using the ROR method to identify statistically significant positive ADR signals associated with PHEs. A total of 99 excipient–ADR pairs, derived from 25 excipients and 51 ADRs, were assessed after excluding pairs with fewer than three reports. Of these, 89 pairs satisfied the pre-specified positive-signal criteria, eight were not statistically significant, and two demonstrated a significantly reduced ROR (Table 6). Among the 99 excipient–ADR associations evaluated, 91 were statistically significant at *P* < 0.05, comprising 89 positive signals with ROR >1 and two protective associations with ROR <1. Following adjustment for the Benjamini–Hochberg false discovery rate (q ≤ 0.05), all 91 significant associations retained their statistical significance (Supplementary Table S2).

**TABLE 6:** Associations between potentially harmful excipients and adverse events.

Adverse drug reactions	No. of reports	ROR (95%CI)	χ^2^	*P*-value
Xylito l(n = 11358)				
Nausea	112	39.81 (32.77, 48.35)	3840.99	**< 0.001**
Vomiting	112	39.81 (32.77, 48.35)	3840.99	**< 0.001**
Diarrhoea	21	3.82 (2.49, 5.88)	43.29	**< 0.001**
Allergic reaction	102	15.75 (12.92, 19.21)	1347.25	**< 0.001**
Aspartame (n = 121605)				
Constipation	9	0.60 (0.31, 1.15)	2.43	0.119
Dizziness	23	1.01 (0.67, 1.52)	0	0.97
Migraine	15	1.09 (0.65, 1.81)	0.1	0.754
Anxiety	6	1.10 (0.49, 2.47)	0.05	0.82
Pruritus	64	1.88 (1.47, 2.42)	25.33	**< 0.001**
Rash	51	2.61 (1.97, 3.47)	47.64	**< 0.001**
Sucralose (n = 6439)				
Diarrhoea	47	15.34 (11.48, 20.49)	612.94	**< 0.001**
Abdominal discomfort	29	23.11 (15.96, 33.46)	592.56	**< 0.001**
Nausea	31	18.14 (12.7, 25.93)	488.09	**< 0.001**
Allergic reaction	73	19.74 (15.63, 24.94)	1251.93	**< 0.001**
Dizziness	23	19.57 (12.93, 29.62)	393.64	**< 0.001**
Acesulfame (n = 1024)				
Allergic reaction	9	14.95 (7.75, 28.84)	115.72	**< 0.001**
Diarrhoea	13	26.50 (15.31, 45.86)	313.20	**< 0.001**
Thimerosal (n = 1358)				
Application site irritation	23	278.35 (181.54, 426.79)	5805.04	**< 0.001**
Allergic reaction	49	63.98 (47.99, 85.29)	2878.43	**< 0.001**
Pruritus	42	115.20 (84.34, 157.35)	4468.40	**< 0.001**
Erythema	26	37.56 (25.43, 55.48)	898.36	**< 0.001**
Methyl Hydroxybenzoate (n = 25441)				
Application site pain	31	12.00 (8.34, 17.25)	293.66	**< 0.001**
Allergic reaction	45	3.01 (2.24, 4.04)	59.32	**< 0.001**
Propylparaben (n = 25441)				
Application site pain	43	17.07 (12.49, 23.32)	595.95	**< 0.001**
Fructose (n = 12267)				
Palpitations	29	52.03 (35.31, 76.65)	1280.01	**< 0.001**
Arrhythmia	10	36.32 (19.0, 69.42)	314.36	**< 0.001**
Nausea	113	37.18 (30.64, 45.13)	3606.35	**< 0.001**
Diarrhoea	84	14.62 (11.75, 18.19)	1020.75	**< 0.001**
Chills	20	76.93 (47.64, 124.24)	1253.18	**< 0.001**
Pyrexia	114	23.58 (19.50, 28.50)	2305.42	**< 0.001**
Sodium Citrate (n = 6298)				
Bradycardia	23	157.59 (100.58, 246.91)	2962.65	**< 0.001**
Vision blurred	6	55.82 (24.37, 127.88)	300.96	**< 0.001**
Nausea	20	11.85 (7.61, 18.44)	195.04	**< 0.001**
Diarrhoea	110	38.16 (31.47, 46.29)	3730.06	**< 0.001**
Constipation	28	38.06 (26.02, 55.67)	958.83	**< 0.001**
Dyspepsia	8	46.11 (22.59, 94.15)	332.77	**< 0.001**
Hypocalcaemia	10	100.34 (51.90, 194.01)	869.11	**< 0.001**
Stress	21	53.47 (34.33, 83.26)	1007.93	**< 0.001**
Cough	29	34.31 (23.63, 49.81)	893.91	**< 0.001**
Saccharin (n = 11296)				
Abdominal discomfort	52	24.18 (18.28, 32.0)	1089.39	**< 0.001**
Allergic reaction	68	10.40 (8.17, 13.24)	560.71	**< 0.001**
Sleep disorder	16	38.68 (23.18, 64.53)	538.02	**< 0.001**
Acetic acid (n = 2328)				
Nausea	27	43.91 (29.93, 64.42)	1096.32	**< 0.001**
Taste disorder	9	145.16 (73.78, 285.61)	1200.41	**< 0.001**
Cough	21	66.75 (43.15, 103.27)	1306.44	**< 0.001**
Pruritus	8	12.14 (6.05, 24.35)	81.01	**< 0.001**
Benzyl Alcohol (n = 894)				
Application site pain	34	393.2 (275.98, 560.21)	11963.35	**< 0.001**
Erythema	38	85.84 (61.88, 119.08)	3005.64	**< 0.001**
Titanium Dioxide (n = 45531)				
Diarrhoea	81	3.75 (3.0, 4.68)	157.29	**< 0.001**
Nausea	62	5.20 (4.03, 6.71)	200.08	**< 0.001**
Dizziness	7	0.82 (0.39, 1.72)	0.28	0.595
Sodium Metabisulfite (n = 1229)				
Erythema	28	44.9 (30.81, 65.44)	1161.59	**< 0.001**
Sorbitol (n = 19163)				
Diarrhoea	122	13.79 (11.49, 16.56)	1364.35	**< 0.001**
Malabsorption	59	18.16 (13.94, 23.66)	889.94	**< 0.001**
Nausea	36	7.07 (5.07, 9.85)	182.11	**< 0.001**
Constipation	83	40.93 (32.44, 51.63)	2772.16	**< 0.001**
Allergic reaction	42	3.73 (2.75, 5.06)	82.56	**< 0.001**
Polyethylene Glycol (n = 116946)				
Eyelid oedema	19	3.82 (2.39, 6.11)	36.13	**< 0.001**
Diarrhoea	98	1.75 (1.43, 2.14)	30.19	**< 0.001**
Nausea	101	3.35 (2.73, 4.10)	153.63	**< 0.001**
Allergic reaction	110	1.61 (1.33,1.94)	24.13	**< 0.001**
Pruritus	79	2.45(1.95, 3.07)	63.85	**< 0.001**
Silicon Dioxide (n = 182219)				
Nausea	35	0.70 (0.50, 0.97)	4.50	**0.034**
Benzalkonium Chloride (n = 15563)				
Eye irritation	125	68.88 (56.72, 83.64)	6802.58	**< 0.001**
Ocular hyperaemia	36	38.50 (27.22, 54.45)	1167.97	**< 0.001**
Eye pain	26	105.39 (67.54, 164.43)	2006.34	**< 0.001**
Vision blurred	16	68.36 (39.79, 117.46)	870.22	**< 0.001**
Eye pruritus	13	66.46 (36.51,120.97)	690.34	**< 0.001**
Propylene Glycol (n = 9957)				
Palpitations	14	28.96 (16.89, 49.68)	356.31	**< 0.001**
Nausea	11	4.08 (2.25, 7.39)	25.36	**< 0.001**
Pruritus	20	7.14 (4.59, 11.10)	103.79	**< 0.001**
Phenol (n = 3452)				
Vomiting	24	26.13 (17.43, 39.19)	565.67	**< 0.001**
Allergic reaction	37	18.43 (13.30, 25.54)	595.71	**< 0.001**
Dizziness	10	15.64 (8.38, 29.19)	135.13	**< 0.001**
Rash	13	23.00 (13.28, 39.82)	268.12	**< 0.001**
Ethanol/Ethyl Alcohol (n = 22351)				
Tachycardia	21	41.40 (25.95, 66.04)	694.53	**< 0.001**
Vertigo	65	37.81 (29.05, 49.22)	1978.83	**< 0.001**
Vomiting	128	23.28 (19.39, 27.94)	2451.36	**< 0.001**
Diarrhoea	89	8.48 (6.86, 10.48)	562.59	**< 0.001**
Application site pain	43	19.44 (14.23, 26.57)	689.14	**< 0.001**
Thirst	12	19.41 (10.75, 35.04)	192.23	**< 0.001**
Injection site swelling	26	30.82 (20.43, 46.48)	656.0	**< 0.001**
Jaundice	43	77.78 (54.89, 110.22)	2394.82	**< 0.001**
Allergic reaction	50	3.82 (2.88, 5.05)	101.91	**< 0.001**
Decreased appetite	15	14.02 (8.31, 23.63)	170.19	**< 0.001**
Somnolence	18	24.24 (14.89, 39.46)	360.46	**< 0.001**
Asphyxia	3	29.55 (8.84, 98.73)	72.81	**< 0.001**
Edetate Disodium (n = 11439)				
Palpitations	3	5.15 (1.65, 16.09)	9.92	**0.002**
Allergic reaction	29	4.30 (2.98, 6.21)	72.65	**< 0.001**
Glycerol (n = 445216)				
Tachycardia	8	0.65 (0.32, 1.32)	1.46	0.227
Vision blurred	10	1.26 (0.65, 2.43)	0.47	0.495
Decreased appetite	49	2.46 (1.80, 3.36)	33.93	**< 0.001**
Lactose (n = 42119)				
Diarrhoea	102	5.16 (4.23, 6.29)	326.13	**< 0.001**
Allergic reaction	34	1.36 (0.97, 1.91)	3.23	0.072
Lactose intolerance	1109	260947.03 (16310.63, 4174782.5)	127007.43	**< 0.001**
Mannitol (n = 173528)				
Diarrhoea	135	1.63 (1.37, 1.94)	31.14	**< 0.001**
Vomiting	162	3.77 (3.19, 4.44)	288.53	**< 0.001**
Allergic reaction	31	0.29 (0.21, 0.42)	52.41	**< 0.001**
Phlebitis	16	4.12 (2.43, 6.97)	32.8	**< 0.001**

ROR, reporting odds ratio; CI, confidence interval. Bold values indicate statistically significant differences (*P* < 0.05).

#### 3.6.1 Local reactions and skin-related events

Benzyl alcohol shown the strongest correlation with injection site pain (ROR = 393.20, *P* < 0.001) and was also significantly associated with erythema (ROR = 85.84, *P* < 0.001). Thiomersal exhibited a strong association with injection site irritation (ROR = 278.35, *P* < 0.001) and pruritus (ROR = 115.20, *P* < 0.001). Sodium metabisulfite was significantly linked to erythema (ROR = 44.90, *P* < 0.001). Benzalkonium chloride exposure was predominantly associated with ocular reactions, including eye pain, eye irritation, blurred vision, and eye pruritus, with respective ROR values of 105.39, 68.88, 68.36, and 66.46 (all *P* < 0.001).

#### 3.6.2 Cardiovascular and systemic events

Sodium citrate was most strongly associated with bradycardia (ROR = 157.59), followed by hypocalcemia (ROR = 100.34). Additionally, it was significantly associated with blurred vision (ROR = 55.82) and stress (ROR = 53.47), with both associations reaching statistical significance (*P* < 0.001).

Fructose was linked to chills, palpitations, nausea, and arrhythmia, with ROR values of 76.93, 52.03, 37.18 and 36.32, respectively (*P* < 0.001). Ethanol exhibited the strongest association with jaundice (ROR = 77.78, *P* < 0.001) and was also significantly associated with tachycardia (ROR = 41.40, *P* < 0.001) and Vertigo (ROR = 37.81, *P* < 0.001).

#### 3.6.3 Gastrointestinal and sensory events

In this study, acetic acid shown the most pronounced association with taste disorder (ROR = 145.16), and was also correlated with cough (ROR = 66.75) and nausea (ROR = 43.91). Xylitol consistently exhibited associations with nausea and vomiting (*P* < 0.001). Sorbitol was mainly associated with constipation (ROR = 40.93, *P* < 0.001) and, to a lesser extent, with malabsorption (ROR = 18.16, *P* < 0.001). Sucralose was significantly linked to abdominal discomfort (*P* < 0.001). Saccharin was associated with sleep disorders, abdominal discomfort, and allergic reaction (*P* < 0.001). Acesulfame exhibited a stronger association with Diarrhoea compared to allergic reaction (*P* < 0.001).

#### 3.6.4 Other system events

The study identified significant associations between phenol and adverse reactions such as rash, vomiting, and allergic reaction. Propylene glycol was exclusively associated with palpitations, evidenced by an ROR of 28.96. Lactose intolerance showed an exceptionally strong association with lactose-containing medications, with 1109 reports in the exposed group compared to none in the non-exposed group. After applying the Haldane-Anscombe correction, the ROR was 260947.03 (*P* < 0.001).

## 4 Discussion

The study provides a comprehensive, multicenter assessment of excipient labeling practices and their correlation with ADRs in pediatric populations in Fujian Province, China. Our study identified variations in excipient labeling information across different hospital types, drug sources, and dosage forms, and established associations between certain excipients and safety signals.

### 4.1 Excipient labeling practices: current status

This study undertook a retrospective analysis of excipient labeling practices across seven medical institutions, revealing substantial disparities in both ingredient and content labeling. The rates of ingredient labeling varied from 24.2% to 61.0%, whereas content labeling was markedly lower, ranging from 0% to 15.7%. An analysis of imported drug labeling based on country of origin revealed considerable geographical variation. Pharmaceutical excipients exhibit both beneficial and detrimental effects, with some posing potential harm. In light of possible adverse reactions, numerous countries have acknowledged the necessity of regulating excipients. Various nations, including the United States, member states of the European Union, Japan, China, and Australia, have established distinct regulatory frameworks governing excipients (Salunke et al., 2017; Saito et al., 2022; Song et al., 2024; Mali et al., 2024; Kinsella et al., 2024). In the United States, the Generally Recognized as Safe (GRAS) list and the Inactive Ingredient Database (IID) serve to identify excipients considered safe for incorporation into drug formulations, accompanied by guidelines for their evaluation. In Europe, the submission of comprehensive dossiers is mandated to secure marketing approval for excipients. China has instituted a dedicated Drug Master File (DMF) system specifically for excipients and has enforced Good Manufacturing Practice (GMP) standards for excipient manufacturers (Mali et al., 2024). Nonetheless, notable discrepancies in excipient labeling practices persist across different countries. The incomplete and inconsistent nature of excipient labeling information imposes numerous constraints on clinical practice (Kinsella et al., 2024). Healthcare professionals encounter challenges in accurately identifying excipient exposure risks from existing labels. For patients with allergies or sensitivities to excipients, physicians must rely on the excipient list provided on the label to assess medication safety. However, the absence or contradiction of labeling information can result in the oversight of excipient exposures that should have been avoided. In the context of incomplete labeling information, the awareness level among healthcare professionals and patients regarding excipient exposure risks is generally low, further complicating clinical decision-making.

### 4.2 Potentially harmful excipient exposure in pediatric populations

The analysis revealed that 41.3% of pediatric cases and 43.2% of all prescriptions included at least one PHE, aligning with international findings (Nellis et al., 2015). Regardless of whether the populations are European or Asian, the prevalence of exposure to potentially harmful excipients remains high, although regional differences in exposure profiles are evident. A multicenter point-prevalence study conducted across 89 neonatal wards in 21 European countries demonstrated that nearly two-thirds of hospitalized neonates received at least one potentially harmful excipient on the day of observation, with parabens being the most frequently used. The exposure rates varied according to geographic region, gestational age, and route of administration (Nellis et al., 2015). Studies conducted in Asia report similar or even higher exposure rates: a Japanese multicenter NICU study found that 82.2% of neonates were exposed to at least one potentially harmful excipient (Saito et al., 2021), while in a Malaysian state hospital, the exposure rate reached 97.2% (Wong et al., 2024). Preterm neonates with very low birth weight and those with a gestational age of less than 30 weeks represent the most significantly exposed populations. Among the former group, 98% were exposed to at least one of the following substances during hospitalization: benzyl alcohol, propylene glycol, or ethanol, with 5%–9% of the administered doses surpassing the recommended levels for adults (Akinmboni et al., 2018). The latter group may encounter exposure to over 20 different types of excipients during their hospital stay (Whittaker et al., 2009). These studies encompass a wide array of time periods and healthcare systems, indicating that excipient exposure is neither a localized nor an occasional issue. Nevertheless, variations in the types of excipients included and the completeness of quantitative data limit the ability to make direct comparisons of exposure rates.

Furthermore, an analysis of usage patterns revealed notable differences between general and specialized hospitals. General hospitals predominantly utilized lactose and mannitol, whereas specialized hospitals more frequently employed antimicrobial preservatives, such as benzyl alcohol, sodium benzoate, ethylparaben, metacresol, and sulfites. The observed differences may be attributed to patient characteristics, disease severity, or institutional prescribing policies. Further investigation is necessary to elucidate the underlying causes.

### 4.3 Safety signals: excipient-associated adverse events

The analysis of the ROR identified several statistically significant safety signals that associate specific excipients with adverse events, many of which align with previously documented toxicities. Propylene glycol, a commonly utilized excipient, raises safety concerns, particularly in critically ill and pediatric populations. This compound undergoes renal excretion (approximately 45%) and hepatic metabolism via alcohol dehydrogenase to lactate (Speth et al., 1987). However, in premature infants and young children, the hepatic and renal functions are not fully developed, leading to a markedly prolonged elimination half-life (ranging from 10.8 to 30.5 h in preterm infants) compared to adults (2–5 h) (De Cock et al., 2014). This disparity indicates a heightened risk of accumulation and potential toxicity. The resultant metabolic bottleneck may trigger a spectrum of dose-dependent toxic effects, including hyperosmolar lactic acidosis, renal impairment, and neurological symptoms (Lim et al., 2014; Burbridge and Jaffe, 2019). Our study identified a correlation between propylene glycol exposure and adverse reactions such as palpitations and nausea, corroborating findings reported in existing literature. While the current study did not allow for a quantitative evaluation of excipient exposure, it is important to highlight that actual clinical exposure levels seem to surpass the established safety thresholds. In a Danish cohort comprising 470 neonates and 160 infants receiving multiple medications, over 70% of cases exceeded the EMA tolerance limit of 50 mg/kg/day for propylene glycol, even when prescriptions containing propylene glycol were considered individually. The maximum daily exposure from all combined drugs reached 954 mg/kg, which is 19.1 times the established limit (Valeur et al., 2018). Similarly, an analysis of Portuguese neonates in neonatal intensive care units using compounded medicines revealed that 75.4% had propylene glycol intake levels surpassing the recommended maximum daily intake (Barbosa et al., 2026). A Japanese multicenter study further indicated that 94.9% of neonates exposed to propylene glycol received daily doses exceeding the acceptable daily intake (ADI), the highest proportion among quantifiable excipients (Saito et al., 2021). In critical care settings, the extent of propylene glycol exceedance is even more concerning; critically ill neonates receiving parenteral drugs via continuous infusion had a median cumulative dose of 4554.5 mg/kg/day, which is approximately 180 times the ADI of 25 mg/kg/day (Shehab et al., 2009). The data collectively indicate that the exceedance of propylene glycol levels is not limited to isolated case reports. For patients receiving extended high-dose infusions, those on multiple medications, or individuals with compromised metabolic or excretory functions (Liamis et al., 2010; Golden et al., 2018; Hayman et al., 2003), it is essential to conduct a risk assessment of cumulative exposure to propylene glycol to mitigate the potential for cumulative toxicity.

Ethanol is a commonly utilized excipient in pediatric formulations, including oral liquids, drops, injectables, and herbal products. Nevertheless, ethanol is not pharmacologically inert, and its safety in pediatric populations—particularly neonates and infants—has garnered increasing scrutiny. Research conducted across various regions has investigated ethanol exposure from pediatric medications employing diverse methodologies, consistently demonstrating widespread exposure and frequent instances of exceeding recommended limits. A community pharmacy survey in Nigeria reported the highest rate of ethanol detection among 380 pediatric oral formulations, with some products potentially surpassing the acceptable daily intake in a single dose (Soremekun et al., 2019). In the Danish SEEN cohort, 38% of neonates and infants were exposed to ethanol as an excipient, and more than half of the ethanol-containing prescriptions exceeded the EMA-recommended daily tolerance limit, with maximum daily exposure reaching 1563 mg/kg/day (Valeur et al., 2018). A retrospective review conducted at a single center in the United States identified 33 ethanol-containing products among 796 medications, of which seven were projected to elevate blood alcohol concentration by more than 2.5 mg/dL at routine doses (Chung et al., 2024). Furthermore, the completeness of labeling remains inadequate; for instance, only 38.8% of ethanol-containing products in Nigeria adhered fully to labeling requirements (Soremekun et al., 2019).

The evidence directly correlating ethanol exposure with specific adverse clinical outcomes remains limited and inconclusive. A single-center retrospective cohort study involving 6,265 hospitalized children under the age of two found no statistically significant association between parenteral ethanol exposure and adverse events (Saito et al., 2024). In contrast, real-world prescription surveys have indicated that ethanol exposure rates and daily doses often exceed recommended limits in neonates and infants undergoing routine pharmacotherapy (Valeur et al., 2018), with younger gestational age being associated with increased accumulation of ethanol metabolites (Zhou et al., 2025). Despite this, current evidence has not established a direct causal relationship between these exposure levels and specific clinical outcomes, such as neurodevelopmental impairment, hypoglycemia, or hepatotoxicity. Against this backdrop, our study provides pharmacovigilance signals from a Chinese inpatient pediatric population. In our ROR analysis, ethanol exposure was associated with jaundice (ROR = 77.78), tachycardia (ROR = 41.40), and vertigo (ROR = 37.81). Although these findings should be regarded as safety signals rather than established causal relationships, they indicate that cumulative daily exposure to ethanol in young children necessitates further investigation, especially considering the prevalent use of ethanol-containing formulations and the vulnerable physiological condition of pediatric patients.

Beyond the two conventional high-risk excipients previously mentioned, our ROR analysis also revealed safety signals for several other pharmaceutical excipients, such as sorbitol, glycerol, and polyethylene glycol, which are consistent with findings from other studies (Saito et al., 2021; Saito et al., 2024). Although these signals are preliminary, they may provide a foundation for further discourse on the safety monitoring of excipients in pediatric drug formulations.

### 4.4 Excipient exposure: risk factors and evidence gaps

Although our ROR analysis identifies statistical associations, it does not establish causality, and residual confounding may be present. In particular, when interpreting excipient-ADR associations, potential confounding by the active pharmaceutical ingredient, concomitant medications, disease severity, and differences in prescribing patterns should be considered, as these factors may influence the observed signals. Exposure patterns in specific populations suggest that excipient exposure is closely linked with disease severity and medical burden. In very low birth weight preterm neonates, conditions such as necrotizing enterocolitis, seizures, bronchopulmonary dysplasia, and extended hospital stay independently predicted increased exposure to benzyl alcohol, propylene glycol, and ethanol (Akinmboni et al., 2018). Furthermore, preterm infants with chronic lung disease exhibited elevated concentrations of ethanol and propylene glycol exposure (Whittaker et al., 2009). The route of administration plays a crucial role; continuous infusion resulted in significantly higher exposure to benzyl alcohol and propylene glycol compared to intermittent administration (Shehab et al., 2009). These factors are intrinsically related to disease severity and polypharmacy, indicating that the burden of excipient exposure is largely a consequence of treatment complexity. This interrelationship implies that observational associations between excipient exposure and disease outcomes are subject to significant confounding risks.

In examining the relationship between excipient exposure and the risk of adverse events, existing evidence predominantly derives from observational studies, rendering the establishment of causality challenging. A retrospective study conducted at a single center revealed that the exposure levels of benzyl alcohol, glycerol, polyethylene glycol, and polysorbates were elevated in individuals experiencing adverse events compared to those without such events (Saito et al., 2024). Another study, which compared critically ill adults, children, and neonates, indicated a potential association between exposure to benzyl alcohol and propylene glycol and an increased risk of mortality, with this association being particularly pronounced in neonates (Sridharan et al., 2020). However, none of these studies utilized robust causal inference methodologies, and the exposure is confounded by factors such as disease severity, polypharmacy, administration route, and monitoring intensity. Consequently, the current body of evidence, characterized by the notion of “high exposure, potential risk,” can only yield suggestive conclusions and does not establish a direct causal link between specific excipient exposure levels and attributable adverse events at the individual level.

### 4.5 Limitations

This study is subject to several limitations that warrant acknowledgment. Firstly, the retrospective design constrains our capacity to establish causal relationships between excipient exposure and adverse events. Secondly, the adverse event data were sourced from the monitoring system of a single hospital, which may not be representative of the broader pediatric population. The underreporting of adverse events is a recognized limitation of passive surveillance systems. Thirdly, the information on excipient content was incomplete for numerous products, which restricts the assessment of dose-response relationships or cumulative exposure. Fourthly, our analysis concentrated on potentially harmful excipients identified through a literature review; other excipients with unknown or emerging safety concerns were not included. Lastly, the study was conducted in Fujian Province, which may limit the generalizability of the findings to other regions of China or countries with different regulatory frameworks.

## 5 Conclusion

In summary, the labeling of excipients in pediatric medications is still inadequate across the institutions examined, exhibiting significant variability based on hospital type, drug source, and dosage form. Hospitalized pediatric patients frequently encounter exposure to potentially harmful excipients. While the ROR analysis identified several safety signals associating specific excipients with adverse drug reactions, the presence of confounding factors cannot be ruled out. Consequently, further research is necessary to validate these findings.

## Data Availability

The raw data supporting the conclusions of this article will be made available by the authors, without undue reservation.
