# Supplementary material for: Potentially harmful excipients in drugs: an analysis of labeling practices and adverse drug reactions
Source: Front Pharmacol. 2026 Oct 13;17:1949057. doi: 10.3389/fphar.2026.1949057 (PMC13710364; doi:10.3389/fphar.2026.1949057)
Supplement: Supplementary file 1 [file Table1.docx]

**Supplementary Table 1.** Summary of adverse events and references for selected potentially harmful excipients.

| **Excipients** | **Category #** | **Adverse events** | **References** |
| --- | --- | --- | --- |
| Xylitol | #1 | Nausea | [1] |
|  | #2 | Vomiting | [1] |
|  | #3 | Diarrhoea | [1] |
|  | #4 | Allergic Reaction | [1,2] |
| Aspartame | #5 | Constipation | [3,4] |
|  | #6 | Dizziness | [3] |
|  | #7 | Migraine | [3] |
|  | #8 | Anxiety | [3] |
|  | #9 | Pruritus | [3] |
|  | #10 | Rash | [3] |
| Sucralose | #3 | Diarrhoea | [3,4] |
|  | #11 | Abdominal Discomfort | [3,4] |
|  | #1 | Nausea | [3,4] |
|  | #4 | Allergic Reaction | [3] |
|  | #6 | Dizziness | [3] |
| Acesulfame | #4 | Allergic Reaction | [3] |
|  | #3 | Diarrhoea | [4] |
| Thimerosal | #12 | Application Site Irritation | [5] |
|  | #4 | Allergic Reaction | [5] |
|  | #9 | Pruritus | [5] |
|  | #13 | Erythema | [5] |
| Methyl Hydroxybenzoate | #14 | Application Site Pain | [6,7] |
|  | #4 | Allergic Reaction | [6,7] |
| Propylparaben | #14 | Application Site Pain | [6,7] |
| Fructose | #15 | Palpitations | [8] |
|  | #16 | Arrhythmia | [8] |
|  | #1 | Nausea | [9] |
|  | #3 | Diarrhoea | [9] |
| Sodium Citrate | #17 | Bradycardia | [10] |
|  | #18 | Vision Blurred | [10] |
|  | #1 | Nausea | [11] |
|  | #3 | Diarrhoea | [11] |
|  | #5 | Constipation | [11] |
|  | #19 | Dyspepsia | [11] |
|  | #20 | Hypocalcaemia | [11] |
|  | #21 | Stress | [11] |
|  | #22 | Cough | [11] |
| Saccharin | #11 | Abdominal Discomfort | [4] |
|  | #4 | Allergic Reaction | [3] |
|  | #23 | Sleep Disorder | [3] |
| Acetic Acid | #1 | Nausea | [12] |
|  | #22 | Cough | [12] |
|  | #9 | Pruritus | [12,13] |
| Benzyl Alcohol | #14 | Application Site Pain | [14] |
|  | #13 | Erythema | [14] |
| Titanium Dioxide | #3 | Diarrhoea | [15] |
|  | #1 | Nausea | [15] |
|  | #6 | Dizziness | [16] |
| Sodium Metabisulfite | #13 | Erythema | [17] |
| Sorbitol | #3 | Diarrhoea | [1] |
|  | #24 | Malabsorption | [1] |
|  | #1 | Nausea | [1] |
|  | #5 | Constipation | [1] |
|  | #4 | Allergic Reaction | [2] |
| Polyethylene Glycol | #25 | Eyelid Oedema | [18] |
|  | #3 | Diarrhoea | [19] |
|  | #1 | Nausea | [19] |
|  | #4 | Allergic Reaction | [18] |
|  | #9 | Pruritus | [18] |
| Silicon Dioxide | #1 | Nausea | [15] |
| Benzalkonium Chloride | #26 | Eye Irritation | [20] |
|  | #27 | Ocular Hyperaemia | [20] |
|  | #28 | Eye Pain | [20] |
|  | #18 | Vision Blurred | [20] |
|  | #29 | Eye Pruritus | [20] |
| Propylene Glycol | #15 | Palpitations | [21] |
|  | #1 | Nausea | [21] |
| Ethanol/Ethyl Alcohol | #30 | Tachycardia | [22,23] |
|  | #31 | Vertigo | [22,23] |
|  | #2 | Vomiting | [22,23] |
|  | #3 | Diarrhoea | [22,23] |
|  | #14 | Application Site Pain | [22,23] |
|  | #32 | Thirst | [23] |
|  | #33 | Injection Site Swelling | [22,23] |
|  | #34 | Jaundice | [22,23] |
|  | #4 | Allergic Reaction | [22,23] |
|  | #35 | Decreased Appetite | [22,23] |
|  | #36 | Somnolence | [22,23] |
|  | #37 | Asphyxia | [22,23] |
| Edetate Disodium | #4 | Allergic Reaction | [24] |
| Glycerol | #35 | Decreased Appetite | [25] |
| Lactose | #3 | Diarrhoea | [26] |
|  | #38 | Lactose Intolerance | [26] |
| Mannitol | #3 | Diarrhoea | [1] |
|  | #2 | Vomiting | [1] |
|  | #4 | Allergic Reaction | [27] |

**References**

[1] Mäkinen KK. Gastrointestinal disturbances associated with the consumption of sugar alcohols with special consideration of xylitol: scientific review and instructions for dentists and other health-care professionals. Int J Dent. 2016; 2016: 5967907.

[2] Cherian P, et al. Safety assessment of mannitol, sorbitol, and xylitol as used in cosmetics. Int J Toxicol. 2025; 44(1_suppl): 22S-43S.

[3] Patel Y, et al. Rediscovering sweetness: the evolution and impact of non-nutritive and natural sweeteners. Curr Nutr Rep. 2025;14(1):54.

[4] Chen HP, et al. Potential effects of low-calorie sweeteners on human health. Nutrients. 2025; 17(16): 2820.

[5] Peng B, et al. Thimerosal induces pseudo-allergic reactions via Mas-related G protein-coupled receptor B2. J Dermatol Sci. 2019; 95(3): 99-106.

[6] Sasseville D. Hypersensitivity to preservatives. Dermatol Ther. 2004;17(3):251-63.

[7] Cashman AL, Warshaw EM. Parabens: a review of epidemiology, structure, allergenicity, and hormonal properties. Dermatitis. 2005; 16(2): 57-66.

[8] Russo E, et al. Fructose and uric acid: major mediators of cardiovascular disease risk starting at pediatric age. Int J Mol Sci. 2020; 21(12): 4479.

[9] Skoog SM, Bharucha AE. Dietary fructose and gastrointestinal symptoms: a review. Am J Gastroenterol. 2004; 99(10): 2046-50.

[10] Khan M, et al.  Propylene glycol neurotoxicity due to sodium citrate therapy in an infant with renal tubular acidosis. Clin Nephrol Case Stud. 2020; 8: 33-36.

[11] Jacobs R, et al. Regional citrate anticoagulation in continuous renal replacement therapy: is metabolic fear the enemy of logic? a systematic review and meta-analysis of randomised controlled trials. Life (Basel). 2023; 13(5): 1198.

[12] Blackley BH, et al. Eye and airway symptoms in hospital staff exposed to a product containing hydrogen peroxide, peracetic acid, and acetic acid. Am J Ind Med. 2023; 66(8): 655-669.

[13] Elhage KG, St. Claire K, Daveluy S. Acetic acid and the skin: a review of vinegar in dermatology. Int J Dermatol. 2022; 61(7): 804-811.

[14] Garg S, et al. Transient pseudophlebitis: Benzyl alcohol-induced transient reactive vasodilation. Indian J Dermatol Venereol Leprol. 2025: 28:1-2.

[15] Yan J, et al. Toxic effects of the food additives titanium dioxide and silica on the murine intestinal tract: mechanisms related to intestinal barrier dysfunction involved by gut microbiota. Environ Toxicol Pharmacol. 2020; 80: 103485.

[16] Zhang X, et al. Neurotoxicity of titanium dioxide nanoparticles: a comprehensive review. Int J Nanomedicine. 2023; 18: 7183-7204.

[17] Vally H, Misso NLA, Madan V. Clinical effects of sulphite additives. Clin Exp Allergy. 2009; 39(11): 1643-51.

[18] Bianchi A, Bottau P, Calamelli E, et al. Hypersensitivity to polyethylene glycol in adults and children: an emerging challenge. Acta Biomed. 2021; 92(S7): e2021519.

[19] Chen SL, et al. Efficacy and complications of polyethylene glycols for treatment of constipation in children: a meta-analysis. Medicine (Baltimore). 2014; 93(16): e65.

[20] Baudouin C, et al. Preservatives in eyedrops: the good, the bad and the ugly. Prog Retin Eye Res. 2010; 29(4): 312-34.

[21] Lim TY, Poole RL, Pageler NM. Propylene glycol toxicity in children. J Pediatr Pharmacol Ther. 2014; 19(4): 277-82.

[22] Mirijello A, et al. Identification and management of acute alcohol intoxication. Eur J Intern Med. 2023; 108: 1-8.

[23] Teschke R. Current View on the Mechanisms of Alcohol-Mediated Toxicity. Int J Mol Sci. 2021; 22(18): 9686.

[24] Ali SB, et al. AstraZeneca ChAdOx1-S COVID-19 vaccine can be safely administered in patients with EDTA allergy. Allergy Asthma Clin Immunol. 2022; 18(1): 22.

[25] Björvell H, Rössner S. Effects of oral glycerol on food intake in man. Am J Clin Nutr. 1982; 36(2): 262-265.

[26] Misselwitz B, Butter M, Verbeke K, et al. Lactose intolerance and malabsorption revisited: exploring the impact and solutions. Dig Dis. 2025; 32(5): 350-7.

[27] Calogiuri GF, et al. Immediate-type hypersensitivity reaction to Mannitol as drug excipient (E421): a case report. Eur Ann Allergy Clin Immunol. 2015; 47(3): 99-102.
