# Supplementary material for: Potentially harmful excipients in drugs: an analysis of labeling practices and adverse drug reactions
Source: Front Pharmacol. 2026 Oct 13;17:1949057. doi: 10.3389/fphar.2026.1949057 (PMC13710364; doi:10.3389/fphar.2026.1949057)
Supplement: Supplementary file 2 [file Table2.docx]

**Supplementary Table 2.** False discovery rate adjustment results for excipient–adverse drug reactions associations.

| **Adverse drug reactions** | **a** | **b** | **c** | **d** | ***P*** | **Critical Value （0.05*i/m）** | **FDR** | **Decision** | |
| --- | --- | --- | --- | --- | --- | --- | --- | --- | --- |
| Xylitol（n=11358） | | | | | | | | |  |
| Nausea | 112 | 11246 | 1214 | 4852245 | 0.00000000E+00 | 9.09000000E-03 | 0.00000000E+00 | Accept | |
| Vomiting | 112 | 11246 | 1214 | 4852245 | 0.00000000E+00 | 8.59000000E-03 | 0.00000000E+00 | Accept | |
| Diarrhoea | 21 | 11337 | 2351 | 4851108 | 4.73877205E-11 | 4.09100000E-02 | 5.79183250E-11 | Accept | |
| Allergic Reaction | 102 | 11256 | 2791 | 4851344 | 7.05886914E-295 | 1.01000000E-02 | 3.49414022E-294 | Accept | |
| Aspartame（n=121605） | | | | | | | | |  |
| Constipation | 9 | 121596 | 589 | 4742623 | 1.19197793E-01 | 4.69700000E-02 | 1.26887973E-01 | Reject | |
| Dizziness | 23 | 121582 | 890 | 4742322 | 9.69914156E-01 | 5.00000000E-02 | 9.69914156E-01 | Reject | |
| Migraine | 15 | 121590 | 539 | 4742673 | 7.53929721E-01 | 4.89900000E-02 | 7.69474663E-01 | Reject | |
| Anxiety | 6 | 121599 | 213 | 4742999 | 8.19996686E-01 | 4.94900000E-02 | 8.28363999E-01 | Reject | |
| Pruritus | 64 | 121541 | 1325 | 4741887 | 4.83630855E-07 | 4.44400000E-02 | 5.44084712E-07 | Accept | |
| Rash | 51 | 121554 | 761 | 4742451 | 5.15192540E-12 | 4.04000000E-02 | 6.37550769E-12 | Accept | |
| Sucralose（n=6439） | | | | | | | | |  |
| Diarrhoea | 47 | 6392 | 2325 | 4849661 | 2.84453504E-135 | 2.12100000E-02 | 6.70497545E-135 | Accept | |
| Abdominal Discomfort | 29 | 6410 | 951 | 4857427 | 7.66102974E-131 | 2.27300000E-02 | 1.68542654E-130 | Accept | |
| Nausea | 31 | 6408 | 1295 | 4857083 | 4.05226445E-108 | 2.52500000E-02 | 8.02348362E-108 | Accept | |
| Allergic Reaction | 73 | 6366 | 2820 | 4855558 | 3.64639510E-274 | 1.21200000E-02 | 1.50413798E-273 | Accept | |
| Dizziness | 23 | 6416 | 890 | 4858378 | 1.44027780E-87 | 2.57600000E-02 | 2.79583338E-87 | Accept | |
| Acesulfame（n=1024） | | | | | | | | |  |
| Allergic Reaction | 9 | 1015 | 2884 | 4860909 | 5.61030934E-27 | 3.53500000E-02 | 7.93458036E-27 | Accept | |
| Diarrhoea | 13 | 1011 | 2359 | 4861434 | 4.67109745E-70 | 2.87900000E-02 | 8.11295873E-70 | Accept | |
| Thimerosal（n=1358） | | | | | | | | |  |
| Application Site Irritation | 23 | 1335 | 301 | 4863158 | 0.00000000E+00 | 2.53000000E-03 | 0.00000000E+00 | Accept | |
| Allergic Reaction | 49 | 1309 | 2844 | 4860615 | 0.00000000E+00 | 3.03000000E-03 | 0.00000000E+00 | Accept | |
| Pruritus | 42 | 1316 | 1347 | 4862112 | 0.00000000E+00 | 3.54000000E-03 | 0.00000000E+00 | Accept | |
| Erythema | 26 | 1332 | 2526 | 4860933 | 2.52112521E-197 | 1.66700000E-02 | 7.56337562E-197 | Accept | |
| Methyl Hydroxybenzoate（n=25441） | | | | | | | | |  |
| Application Site Pain | 31 | 25410 | 492 | 4838884 | 8.40760322E-66 | 2.98000000E-02 | 1.41076732E-65 | Accept | |
| Allergic Reaction | 45 | 25396 | 2848 | 4836528 | 1.35109588E-14 | 3.93900000E-02 | 1.71485246E-14 | Accept | |
| Propylparaben（n=25441） | | | | | | | | |  |
| Application Site Pain | 43 | 25398 | 480 | 4838896 | 1.40687591E-131 | 2.17200000E-02 | 3.23908641E-131 | Accept | |
| Fructose（n=12267） | | | | | | | | |  |
| Palpitations | 29 | 12238 | 221 | 4852329 | 2.88231559E-280 | 1.11100000E-02 | 1.29704202E-279 | Accept | |
| Arrhythmia | 10 | 12257 | 109 | 4852441 | 2.61541514E-70 | 2.82800000E-02 | 4.62368033E-70 | Accept | |
| Nausea | 113 | 12154 | 1213 | 4851337 | 0.00000000E+00 | 5.10000000E-04 | 0.00000000E+00 | Accept | |
| Diarrhoea | 84 | 12183 | 2288 | 4850262 | 6.31238397E-224 | 1.51500000E-02 | 2.08308671E-223 | Accept | |
| Chills | 20 | 12247 | 103 | 4852447 | 1.94501947E-274 | 1.16200000E-02 | 8.37204031E-274 | Accept | |
| Pyrexia | 114 | 12153 | 1930 | 4850620 | 0.00000000E+00 | 1.01000000E-03 | 0.00000000E+00 | Accept | |
| Sodium Citrate（n=6298） | | | | | | | | |  |
| Bradycardia | 23 | 6275 | 113 | 4858406 | 0.00000000E+00 | 1.52000000E-03 | 0.00000000E+00 | Accept | |
| Vision Blurred | 6 | 6292 | 83 | 4858436 | 2.16819292E-67 | 2.92900000E-02 | 3.70088101E-67 | Accept | |
| Nausea | 20 | 6278 | 1306 | 4857213 | 2.63976845E-44 | 3.18200000E-02 | 4.14820756E-44 | Accept | |
| Diarrhoea | 110 | 6188 | 2262 | 4856257 | 0.00000000E+00 | 6.57000000E-03 | 0.00000000E+00 | Accept | |
| Constipation | 28 | 6270 | 570 | 4857949 | 1.81499337E-210 | 1.61600000E-02 | 5.61513573E-210 | Accept | |
| Dyspepsia | 8 | 6290 | 134 | 4858385 | 2.56777829E-74 | 2.72700000E-02 | 4.70759352E-74 | Accept | |
| Hypocalcaemia | 10 | 6288 | 77 | 4858442 | 5.74077422E-191 | 1.86900000E-02 | 1.53604499E-190 | Accept | |
| Stress | 21 | 6277 | 304 | 4858215 | 3.85945251E-221 | 1.56600000E-02 | 1.23253483E-220 | Accept | |
| Cough | 29 | 6269 | 655 | 4857864 | 2.34396710E-196 | 1.71700000E-02 | 6.82508068E-196 | Accept | |
| Saccharin（n=11296） | | | | | | | | |  |
| Abdominal Discomfort | 52 | 11244 | 928 | 4852899 | 7.65062791E-239 | 1.46500000E-02 | 2.61176608E-238 | Accept | |
| Allergic Reaction | 68 | 11228 | 2825 | 4850696 | 6.49804836E-124 | 2.42400000E-02 | 1.34022247E-123 | Accept | |
| Sleep Disorder | 16 | 11280 | 178 | 4853343 | 5.56883105E-119 | 2.47500000E-02 | 1.12513117E-118 | Accept | |
| Acetic Acid（n=2328） | | | | | | | | |  |
| Nausea | 27 | 2301 | 1299 | 4861190 | 2.38524596E-240 | 1.41400000E-02 | 8.43354821E-240 | Accept | |
| Taste Disorder | 9 | 2319 | 130 | 4862359 | 5.70758250E-263 | 1.26300000E-02 | 2.26020267E-262 | Accept | |
| Cough | 21 | 2307 | 663 | 4861826 | 5.20525412E-286 | 1.06100000E-02 | 2.45390552E-285 | Accept | |
| Pruritus | 8 | 2320 | 1381 | 4861108 | 2.27634369E-19 | 3.73700000E-02 | 3.04537872E-19 | Accept | |
| Benzyl Alcohol（n=894） | | | | | | | | |  |
| Application Site Pain | 34 | 860 | 489 | 4863434 | 0.00000000E+00 | 7.07000000E-03 | 0.00000000E+00 | Accept | |
| Erythema | 38 | 856 | 2514 | 4861409 | 0.00000000E+00 | 5.56000000E-03 | 0.00000000E+00 | Accept | |
| Titanium Dioxide（n=45531） | | | | | | | | |  |
| Diarrhoea | 81 | 45450 | 2291 | 4816995 | 4.58183512E-36 | 3.38400000E-02 | 6.77017428E-36 | Accept | |
| Nausea | 62 | 45469 | 1264 | 4818022 | 2.09637303E-45 | 3.13100000E-02 | 3.34743435E-45 | Accept | |
| Dizziness | 7 | 45524 | 906 | 4818380 | 5.95369990E-01 | 4.84800000E-02 | 6.13975302E-01 | Reject | |
| Sodium Metabisulfite（n=1229） | | | | | | | | |  |
| Erythema | 28 | 1201 | 2524 | 4861064 | 1.55705019E-254 | 1.36400000E-02 | 5.70918404E-254 | Accept | |
| Sorbitol（n=19163） | | | | | | | | |  |
| Diarrhoea | 122 | 19041 | 2250 | 4843404 | 1.35837034E-298 | 9.60000000E-03 | 7.07782440E-298 | Accept | |
| Malabsorption | 59 | 19104 | 824 | 4844830 | 1.70412211E-195 | 1.76800000E-02 | 4.82023112E-195 | Accept | |
| Nausea | 36 | 19127 | 1290 | 4844364 | 1.74357682E-41 | 3.28300000E-02 | 2.65560162E-41 | Accept | |
| Constipation | 83 | 19080 | 515 | 4845139 | 0.00000000E+00 | 4.04000000E-03 | 0.00000000E+00 | Accept | |
| Allergic Reaction | 42 | 19121 | 2851 | 4842803 | 1.03871864E-19 | 3.68700000E-02 | 1.40867322E-19 | Accept | |
| Polyethylene Glycol（n=116946） | | | | | | | | |  |
| Eyelid Oedema | 19 | 116927 | 202 | 4747669 | 1.84877951E-09 | 4.14100000E-02 | 2.23206306E-09 | Accept | |
| Diarrhoea | 98 | 116848 | 2274 | 4745597 | 3.92559046E-08 | 4.34300000E-02 | 4.51899367E-08 | Accept | |
| Nausea | 101 | 116845 | 1225 | 4746646 | 2.87959684E-35 | 3.43400000E-02 | 4.19235423E-35 | Accept | |
| Allergic Reaction | 110 | 116836 | 2783 | 4745088 | 9.03307387E-07 | 4.49500000E-02 | 1.00480260E-06 | Accept | |
| Pruritus | 79 | 116867 | 1310 | 4746561 | 1.35401803E-15 | 3.88900000E-02 | 1.74088032E-15 | Accept | |
| Silicon Dioxide（n=182219） | | | | | | | | |  |
| Nausea | 35 | 182184 | 1291 | 4681307 | 3.38709090E-02 | 4.59600000E-02 | 3.68485713E-02 | Accept | |
| Benzalkonium Chloride（n=15563） | | | | | | | | |  |
| Eye Irritation | 125 | 15438 | 570 | 4848684 | 0.00000000E+00 | 5.05000000E-03 | 0.00000000E+00 | Accept | |
| Ocular Hyperaemia | 36 | 15527 | 292 | 4848962 | 6.40425781E-256 | 1.31300000E-02 | 2.43854432E-255 | Accept | |
| Eye Pain | 26 | 15537 | 77 | 4849177 | 0.00000000E+00 | 4.55000000E-03 | 0.00000000E+00 | Accept | |
| Vision Blurred | 16 | 15547 | 73 | 4849181 | 3.30644273E-191 | 1.81800000E-02 | 9.09271751E-191 | Accept | |
| Eye Pruritus | 13 | 15550 | 61 | 4849193 | 4.19790136E-152 | 1.97000000E-02 | 1.06562111E-151 | Accept | |
| Propylene Glycol（n=9957） | | | | | | | | |  |
| Palpitations | 14 | 9943 | 236 | 4854624 | 1.92387212E-79 | 2.67700000E-02 | 3.59364792E-79 | Accept | |
| Nausea | 11 | 9946 | 1315 | 4853545 | 4.77236734E-07 | 4.39400000E-02 | 5.43062490E-07 | Accept | |
| Pruritus | 20 | 9937 | 1369 | 4853491 | 2.30132454E-24 | 3.58600000E-02 | 3.20888915E-24 | Accept | |
| Phenol（n=3452） | | | | | | | | |  |
| Vomiting | 24 | 3428 | 1302 | 4860063 | 5.40706684E-125 | 2.32300000E-02 | 1.16369482E-124 | Accept | |
| Allergic Reaction | 37 | 3415 | 2856 | 4858509 | 1.58146894E-131 | 2.22200000E-02 | 3.55830511E-131 | Accept | |
| Dizziness | 10 | 3442 | 903 | 4860462 | 3.18860975E-31 | 3.48500000E-02 | 4.57496181E-31 | Accept | |
| Rash | 13 | 3439 | 799 | 4860566 | 3.08255355E-60 | 3.08100000E-02 | 5.00283281E-60 | Accept | |
| Ethanol/Ethyl Alcohol（n=22351） | | | | | | | | |  |
| Tachycardia | 21 | 22330 | 110 | 4842356 | 5.15550912E-153 | 1.91900000E-02 | 1.34314580E-152 | Accept | |
| Vertigo | 65 | 22256 | 374 | 4842122 | 0.00000000E+00 | 6.06000000E-03 | 0.00000000E+00 | Accept | |
| Vomiting | 128 | 22223 | 1198 | 4841268 | 0.00000000E+00 | 2.02000000E-03 | 0.00000000E+00 | Accept | |
| Diarrhoea | 89 | 22262 | 2283 | 4840183 | 2.52940879E-124 | 2.37400000E-02 | 5.32790363E-124 | Accept | |
| Application Site Pain | 43 | 22308 | 480 | 4841986 | 7.67239275E-152 | 2.02000000E-02 | 1.89891721E-151 | Accept | |
| Thirst | 12 | 22339 | 134 | 4842332 | 1.07845600E-43 | 3.23200000E-02 | 1.66823662E-43 | Accept | |
| Injection Site Swelling | 26 | 22325 | 183 | 4842283 | 1.23085157E-144 | 2.07100000E-02 | 2.97205622E-144 | Accept | |
| Jaundice | 43 | 22308 | 120 | 4842346 | 0.00000000E+00 | 8.08000000E-03 | 0.00000000E+00 | Accept | |
| Allergic Reaction | 50 | 22301 | 2843 | 4839623 | 5.93330644E-24 | 3.63600000E-02 | 8.15829636E-24 | Accept | |
| Decreased Appetite | 15 | 22336 | 232 | 4842234 | 6.95968695E-39 | 3.33300000E-02 | 1.04395304E-38 | Accept | |
| Somnolence | 18 | 22333 | 161 | 4842305 | 2.40043723E-80 | 2.62600000E-02 | 4.57006319E-80 | Accept | |
| Asphyxia | 3 | 22348 | 22 | 4842444 | 1.44858288E-17 | 3.78800000E-02 | 1.91212941E-17 | Accept | |
| Edetate Disodium（n=11439） | | | | | | | | |  |
| Palpitations | 3 | 11436 | 247 | 4853131 | 1.63338300E-03 | 4.54500000E-02 | 1.79672130E-03 | Accept | |
| Allergic Reaction | 29 | 11410 | 2864 | 4850514 | 1.56976913E-17 | 3.83800000E-02 | 2.04483084E-17 | Accept | |
| Glycerol（n=445216） | | | | | | | | |  |
| Tachycardia | 8 | 445208 | 123 | 4419478 | 2.26796388E-01 | 4.74700000E-02 | 2.38860025E-01 | Reject | |
| Vision Blurred | 10 | 445206 | 79 | 4419522 | 4.95294243E-01 | 4.79800000E-02 | 5.16148737E-01 | Reject | |
| Decreased Appetite | 49 | 445167 | 198 | 4419403 | 5.73821771E-09 | 4.19200000E-02 | 6.84438016E-09 | Accept | |
| Lactose（n=42119） | | | | | | | | |  |
| Diarrhoea | 102 | 42017 | 2270 | 4820438 | 7.15391878E-73 | 2.77800000E-02 | 1.28770538E-72 | Accept | |
| Allergic Reaction | 34 | 42085 | 2859 | 4819839 | 7.23053078E-02 | 4.64600000E-02 | 7.78067986E-02 | Reject | |
| Lactose Intolerance | 1109.5 | 41010.5 | 0.5 | 4822698.5 | 0.00000000E+00 | 7.58000000E-03 | 0.00000000E+00 | Accept | |
| Mannitol（n=173528） | | | | | | | | |  |
| Diarrhoea | 135 | 173393 | 2237 | 4689052 | 2.41133229E-08 | 4.29300000E-02 | 2.80849290E-08 | Accept | |
| Vomiting | 162 | 173366 | 1164 | 4690125 | 1.10589457E-64 | 3.03000000E-02 | 1.82472604E-64 | Accept | |
| Allergic Reaction | 31 | 173497 | 2862 | 4688427 | 4.54817510E-13 | 3.99000000E-02 | 5.69961183E-13 | Accept | |
| Phlebitis | 16 | 173512 | 105 | 4691184 | 1.02442474E-08 | 4.24200000E-02 | 1.20735772E-08 | Accept | |

FDR, false discovery rate. The symbols a, b, c, and d represent the number of patient–ADR observations within each cell of the 2 × 2 table, with each patient counted once per excipient–ADR pair. 'a' denotes the number of reports containing both the target drug and the target adverse reactions; 'b' represents the number of reports containing the target drug with other ADRs; 'c' indicates the number of reports containing other drugs with the target ADRs; and 'd' signifies the number of reports containing both other drugs and other ADRs
